# An *in vitro* study to determine the sealing ability of sealers with and without smear layer removal

**DOI:** 10.4103/0972-0707.58335

**Published:** 2009

**Authors:** Swaty Jhamb, Vineeta Nikhil, Vijay Singh

**Affiliations:** Department of Conservative and Endodontics, Dr. H.S. Institute of Dental Sciences, Sector-25, P.U, Chandigarh, India; 1 Department of Conservative and Endodontics, D. A. V (C) Dental College, Yamuna Nagar, Haryana, India

**Keywords:** Acroseal, microleakage, stereomicroscope, Ketac-Endo

## Abstract

**Aims/Objectives:**

The objective of this *in vitro* study is to compare the sealing ability of Ketac-Endo and Acroseal.

**Materials and Methods:**

Seventy teeth were selected and sectioned at the cementoenamel junction. The teeth were cleaned and shaped, and they were divided into five different groups. Group1: 20 teeth in which the smear layer was left intact using sodium hypochlorite. Group 2: 20 teeth in which the smear layer was removed using 17% EDTA. Group 3: 20 teeth in which the smear layer was removed using 17% EGTA. These groups (1, 2, and 3) were further subdivided into two subgroups (a and b) by obturation utilizing lateral condensation with Gutta-percha and Acroseal (subgroup “a”) or Ketac-Endo (subgroup “b”). The subgroups contained 10 teeth each. Group 4: 5 teeth that were instrumented but not obturated. Group 5:5 teeth that were neither instrumented nor obturated. The specimens were immersed in methylene blue dye, and microleakage was assessed using a stereomicroscope.

**Results:**

The data was analyzed using one way analysis of variance and student's *t*-test.

**Conclusion:**

17% EGTA is a better and potent alternative to 17% EDTA for smear layer removal. Acroseal sealer has less microleakage as compared with Ketac-Endo. Sealing ability of Acroseal sealer is better when it is used in conjunction with 17% EGTA.

## INTRODUCTION

The major aim of endodontic therapy is to attain a clean canal that allows for the three-dimensional obturation of root canal system along with a hermetic sealing. During instrumentation, a smear layer is found, which is a thin layer that occludes the orifices of the dentinal tubules and covers the inter tubular dentin of the prepared root canal. The smear layer consists of organic and inorganic substances, which include fragments of odontoblastic processes, microorganisms, and necrotic materials. This layer cannot be seen using the naked eye. It consists of a superficial layer on the surface of canal wall, which is approximately 1 to 2 microns in thickness and a deeper layer packed into the dentinal tubules to a depth of 40 microns. Presence of this smear layer prevents the penetration of intracanal medication into the irregularities of the root canal system and the dentinal tubules and also prevents the complete adaptation of obturation materials to the prepared root canal surfaces. Various chemicals have been used to remove the smear layer. For this, 17% EGTA and 17% EDTA are used. Both the solutions have a strong demineralizing effect; they widen the apertures of dentinal tubules, soften the dentin and remove the smear layer and smear plugs at the tubular apertures. The removal of smear layer is followed by obturation of the root canal. The most commonly used obturation material is Gutta-percha with a sealer. Gutta-percha serves as the core-filling material, whereas the sealer acts as a binding agent. In addition, the sealer fills in the discrepancies between the individual Gutta-percha cones and between the cones and the dentinal walls. It also serves as a lubricant for the easier placement of the core material. This allows the tooth and material to act as a single unit reducing any leakage.

The main purpose of this study was to compare the microleakage of Acroseal and Ketac-Endo with and without smear layer. The smear layer was removed using 17% EDTA and 17% EGTA, and the smear layer was retained in samples using 5% sodium hypochlorite.

## MATERIALS AND METHODS

For the *in vitro* study, 70 extracted maxillary central Incisors from patients in the age group 40-55 years were obtained from the Department of Oral and Maxillofacial Surgery, D. A. V. (C) Dental College, Yamuna Nagar, Haryana. After extraction, soft tissue and calculus were mechanically removed, and teeth were stored in 5% sodium hypochlorite solution for 24 h to remove any remaining soft tissue. The teeth were sectioned at the cementoenamel junction using a diamond disc and water spray The sectioned teeth were taken and a working length for each root was then established 1 mm short of the apical foramen using a No. 20 K-file and then roots were divided randomly into three groups.

### Experimental groups

#### Group I

Twenty roots were taken and a standardized technique described by Grossman was used to instrument each root canal three sizes larger than the first file that bound at the established working length. In these, the smear layer was left intact and irrigation was done using 15 ml of 5% sodium hypochlorite solution.

#### Group II

Twenty teeth were prepared as described in Group 1, and the smear layer was removed using 10 ml of 17% EDTA solution, followed by a final irrigation with 10 ml of 5% sodium hypochlorite.

#### Group III

Twenty teeth were prepared as described in Group 1 and Group 2, and the smear layer was removed using 10 ml of 17% EGTA, followed by a final irrigation with 10 ml of 5% sodium hypochlorite.

These groups were further subdivided into two subgroups (a and b) by obturation utilizing lateral condensation with Gutta-percha and either Acroseal (subgroup “a”) or Ketac Endo (subgroup “b”). The subgroups contained 10 teeth each.

#### Group IV (positive control)

Five roots were taken and instrumented using standardized technique but were not obturated.

#### Group V (negative control)

Five roots were taken and were neither instrumented nor obturated.

The roots were coronally sealed with Cavit. The specimens were placed in cotton moistened with sterile water to attain 100% humidity and then were placed in an incubator at 37°C for 8 days to allow the sealers to set completely. After 8 days, each root was blotted dry and then covered with two coats of nail polish, except for the apical 2 mm. The negative control group was fully covered with nail polish. The nail polish was allowed to air dry for 24 h. Experimental and control roots were suspended in aqueous 1% methylene blue dye. After 6 days, the roots were removed from the dye, rinsed with saline and longitudinally sectioned with a chisel and mallet after placing two grooves, one buccally and one lingually. Each section was then viewed under a stereomicroscope. Linear apical leakage was measured from the apex to the most coronal extent of dye penetration. A calibrated reticle (0.01) mm was used. Stereomicroscope was used to facilitate a magnified and clear view along with an accuracy of 0.01 mm.

The results were obtained and subjected to statistical analysis using one way analysis of variance and unpaired student's ‘*t*’ test.

## RESULTS

The mean microleakage values of Group 1a were 4.04 mm, Group 1b – 6.19 mm, Group 2a – 3.03 mm, Group 2b – 3.83 mm, Group 3a – 1.97 mm, Group 3b – 2.54 mm, positive control – 8.95 mm and negative control – 0.43 mm [Tables 1 and 2].

**Table 1:** Mean values of microleakage

Groups	Mean microleakage values (mm)
1a	4.04
1b	6.19
2a	3.03
2b	3.83
3a	1.97
3b	2.54
4	8.95
5	0.43

a = acroseal, b = Ketac Endo

**Table 2:** Student's ‘*t*’-test values

Groups	‘*t*’ values	Status
1a - 1b	5.596	0.00003
1a - 2a	2.189	0.04202
1a - 2b	0.513	0.61419
1a - 3a	5.391	0.00004
1a - 3b	4.071	0.00072
1a - 4	9.440	0.00000
1a -5	7.252	0.00001
1b - 2a	8.773	0.00000
1b - 2b	8.009	0.00000
1b - 3a	16.429	0.0000
1b - 3b	15.695	0.0000
1b - 4	8.609	0.00000
1b - 5	20.447	0.00000
2a - 2b	2.053	0.05490
2a - 3a	2.964	0.00831
2a - 3b	1.440	0.16703
2a - 4	13.472	0.00000
2a - 5	5.693	0.00007
2b - 3a	6.328	0.0000
2b - 3b	4.722	0.00017
2b - 4	13.472	0.00000
2b - 5	9.765	0.00000
3a - 3b	2.478	0.02335
3a - 4	21.965	0.00000
3a - 5	5.546	0.00009
3b - 4	23.212	0.00000
3b - 5	9.216	0.00000
4 - 5	27.770	0.00000

a = acroseal, b = Ketac Endo, *P* < 0.05 (significant), *P* > 0.05(insignificant)

## DISCUSSION

By comparing the microleakage of Acroseal (Septodont) with Ketac-Endo (3M), the study attempted to standardize the testing conditions and wherever possible to mimic the oral environment. A temperature of 37°C and incubation period of 8 days was given to allow sealers to set as in clinical situations. Methylene blue dye was chosen as an indicator dye.[1] The penetration of dye was judged and correct readings were obtained by using the stereomicroscope.

While comparing the microleakages between the groups using 5% sodium hypochlorite and those using 17% EDTA and 17% EGTA, the microleakage in the former were found to be the highest. The possible reason for this result may be that 5% sodium hypochlorite does not remove the smear layer but only flushes out the organic debris. Hence, there is a tendency for greater infiltration of dye. This is because the smear layer is considered as an interface between the obturating sealer and the dentinal wall. The smear layer is an amorphous, non-homogeneous, weakly adherent structure and has a low density because of its higher water content, which makes it unstable and hence susceptible to dye infiltration. 17% EDTA and 17% EGTA removed the smear layer completely.[3] 17% EDTA is a chelator and has the capacity to completely remove the smear layer, cause enlargement of dentinal tubules, softening of the dentin and denaturation of the collagen fibers. The tubular orifices of instrumented canals were enlarged. 17% EGTA efficiently removed the smear layer without inducing erosive action and allowed the sealers to penetrate the dentinal tubules completely, thereby ensuring a good seal with minimum microleakage.[3] The removal of smear layer eliminates its disadvantages, and the obturating material is allowed to have a very close contact with the dentin wall. Additionally, the opened dentinal tubules permit increased surface contact between the dentin and the sealer, which could improve the impermeability of the obturation.[4] The removal of smear layer allowed the root canal sealer to contact the canal wall and completely penetrate the dentinal walls. This ensures a tight seal, thereby reducing the amount of microleakage.[2]

Teeth that were instrumented but not obturated displayed the highest amount of microleakage. The reason could be that there was no obturating material present to seal the instrumented root canal. This allowed complete dye penetration.

Teeth that were neither instrumented nor obturated retained a good amount of healthy and sound tooth structure; therefore, the leakage was negligible.

When the microleakage readings of groups using 17% EDTA were compared with groups using 17% EGTA, the values were found to be higher in the former group. The reason may be that 17% EGTA has a greater potency as a chelator than 17% EDTA. 17% EGTA has an ability to efficiently remove smear layer, whereas 17% EDTA not only removes the smear layer, but also causes excessive dissolution of peritubular and intertubular dentin along with its erosion.[3] This erosive property of 17% EDTA is responsible for the greater penetration of dye. In contrast, the good chelating ability of 17% EGTA ensures a greater depth of penetration of sealers into the dentinal tubules and reduces any chance of gap formation. This allows a hermetic sealing of the root canal.[4]

The sealing ability of groups using Ketac-Endo is higher in comparison to groups using Acroseal. This is because Ketac-Endo is a technique-sensitive material. The material is sensitive to moisture. Ketac-Endo may not have set properly. Nail polish that was left to air dry for 24 h, which might have led to the dehydration of the sealer. The ability of sealer to flow seemed to diminish between 7 min and 13 min. Also, the rotation of the spreader and the time needed for lateral condensation is a detriment to seal provided by Ketac-Endo. Ketac-Endo shows poor adhesion to Gutta-percha. Moreover, Ketac-Endo shows higher setting shrinkage of 3.6 vol.%, which results in adhesive failure as a result of contraction stress.[1,5–7] 17% EDTA and 17% EGTA might have chemically or physically altered the sealer Ketac-Endo in apical region. This might have disturbed the complete adhesion of Ketac-Endo to dentinal tubules. Further, this could have caused greater leakage as it is mentioned that dye penetrates more easily through adhesive failures at the dentin interface in restorations.[8]

Low microleakage in Acroseal groups is due to the property of Acroseal that ensures adequate adhesion of Gutta-percha points to canal walls in providing total sealing. It also has a capacity to bind Gutta- percha points together (Septodont).

## CONCLUSION

1. 17% EGTA is a better and potent alternative to 17% EDTA for smear layer removal.
2. Acroseal sealer has less microleakage as compared to Ketac-Endo.
3. Sealing ability of Acroseal sealer is better when it is used in conjunction with 17% EGTA.
